# Biomechanical comparison of headless compression screws versus independent locking screw for intra-articular fractures

**DOI:** 10.1007/s00590-023-03792-8

**Published:** 2023-12-19

**Authors:** Meghana Mandala, Shalin Shaunak, Paul Kreitmair, Joideep Phadnis, Enis Guryel

**Affiliations:** 1 Brighton and Sussex Medical School, Brighton, UK; 2 University Hospitals Sussex NHS Foundation Trust, Brighton, UK; 3 University of Sussex, Brighton, UK

**Keywords:** Intra-articular fractures, Headless compression screws, Locking screws, Acutrak, Synthes, Smith and Nephew, Compression force

## Abstract

**Purpose:**

Headless compression screws (HCS) have a variable thread pitch and headless design enabling them to embed below the articular surface and generate compression force for fracture healing without restricting movement. Locking screws have greater variety of dimensions and a threaded pitch mirroring the design of the HCS. The objective of this study is to determine whether locking screws can generate compression force and compare the compressive forces generated by HCS versus locking screws.

**Method:**

A comparison between 3.5-mm HCS versus 3.5-mm locking screws and 2.8-mm HCS versus 2.7-mm locking screws was performed using a synthetic foam bone model (Synbone) and FlexiForce sensors to record the compression forces (*N*). The mean peak compression force was calculated from a sample of 3 screws for each screw type. Statistical analysis was performed using the one-way ANOVA test and statistical significance was determined to be *p* =  < *0.05.*

**Results:**

The 3.5-mm Synthes and Smith and Nephew locking screws generated similar peak compression forces to the 3.5-mm Acutrak 2 headless compression screws with no statistically significant difference between them. The smaller 2.7-mm Synthes and Smith and Nephew locking screws initially generated similar compressive forces up to 1.5 and 2 revolutions, respectively, but their peak compression force was less compared to the 2.8-mm Micro Acutrak 2 HCS.

**Conclusion:**

Locking screws are able to generate compressive forces and may be a viable alternative to headless compressive screws supporting their use for intra-articular fractures.

**Supplementary Information:**

The online version contains supplementary material available at 10.1007/s00590-023-03792-8.

## Introduction

Headless compression screws (HCS) are used for the internal fixation of intra-articular fractures, such as fractures of the scaphoid, capitellum and talus [1]. Compared to traditional screws, HCSs are favoured as their design enables them to be embedded below the articular surface of bone in order to generate a compressive force across the fracture without restricting movement. Interfragmentary compression is important to facilitate stability, allow early rehabilitation and better union rates [2, 3].

The HCS was developed by Herbert in 1984 [4] and modified by Whipple by developing a cannulated version [1]. These screws have another set of threads in place of the screw head. The pitch of these threads on the head is finer than those on the leading edge thereby generating compression across the fracture line. Whilst these screws have proven to be popular in the internal fixation of intra-articular fractures, notable drawbacks included the surgical exposure required, poor compressive forces and lack of versatility of screw specifications [5, 6]. The second generation of HCS was designed to allow greater compression [1] and cannulated to allow percutaneous fixation. The Acutrak screw (Acumed LLC, Hillsboro, Oregon) is a tapered cannulated HCS with a variable pitch along the full length of the screw [7]. Other second-generation screws evolved on the principles of increased compressive strength and improved versatility such as the CAPTIVATE HCS (Globus Medical Inc, Audubon, Pennsylvania) [8] and Synthes HCS (DePuy Synthes, West Chester, Pennsylvania) [1, 5, 9]. These second-generation HCSs allow for good compressive forces and percutaneous fixation which is important for smaller intra-articular fractures, such as fractures of the scaphoid [3, 10, 11].

Our observation is that the design of locking screws is similar to HCSs. Locking screws have threads on the screw head which allow them to be fixed to corresponding threads in a locking plate [9, 12–14]. The thread on the head of a locking screw has a smaller pitch compared to the shaft (tip) of the screw. This is to enable the screw head to engage with the threads within the locking plate hole. This gives the screw similar properties to that of a headless compression screw. Locking screws are used in most hospitals and there is often a large array of diameters and lengths available and thus may be used across a variety of intra-articular fracture patterns and clinical scenarios. Furthermore, these locking screws are much more cost-effective in comparison with headless compression screws.

### Objective

Our hypothesis is that locking screws generate comparable compressive forces to HCSs. Previous studies have been conducted comparing various HCSs available [1, 3, 8, 11, 15–17] and a previous study by Felstead et al. [12] demonstrated that locking screws can be used for intra-articular elbow fractures. However, biomechanical studies have not been conducted comparing the compression forces generated between a second-generation HCS and an independent locking screw of similar specifications. The objective of this study is to compare the compression force generated by locking screws and HCS in a simulated intra-articular fracture.

## Materials and methods

### Materials

The HCSs used within the study include the micro Acutrak and mini Acutrak screws (Acumed, Oregon, USA), Smith and Nephew 3.5-mm and 2.7-mm fully threaded EVOS locking screws (Smith and Nephew Inc, Memphis, USA) and Synthes 2.7-mm and 3.5-mm locking screws (DePuy Synthes, USA). These screws were chosen for their similar dimensions to the selected headless compression screws.

The study was conducted using synthetic polyurethane foam bone models (Synbone AG, Switzerland), which demonstrate compressive strength of 2–4 MPa and hold similar physical properties to trabecular bone. Block A measured 75 mm × 100 mm × 13 mm and separated by four FlexiForce Standard A201 pressure sensors (Tekscan Inc, Boston, USA) from block B which measured 75 mm × 100 mm × 37 mm. As the screws were tightened the number of revolutions of the screw was recorded together with the compression forces generated by each screw at each revolution.

### Method

#### Sensor calibration

**Fig. 1:**
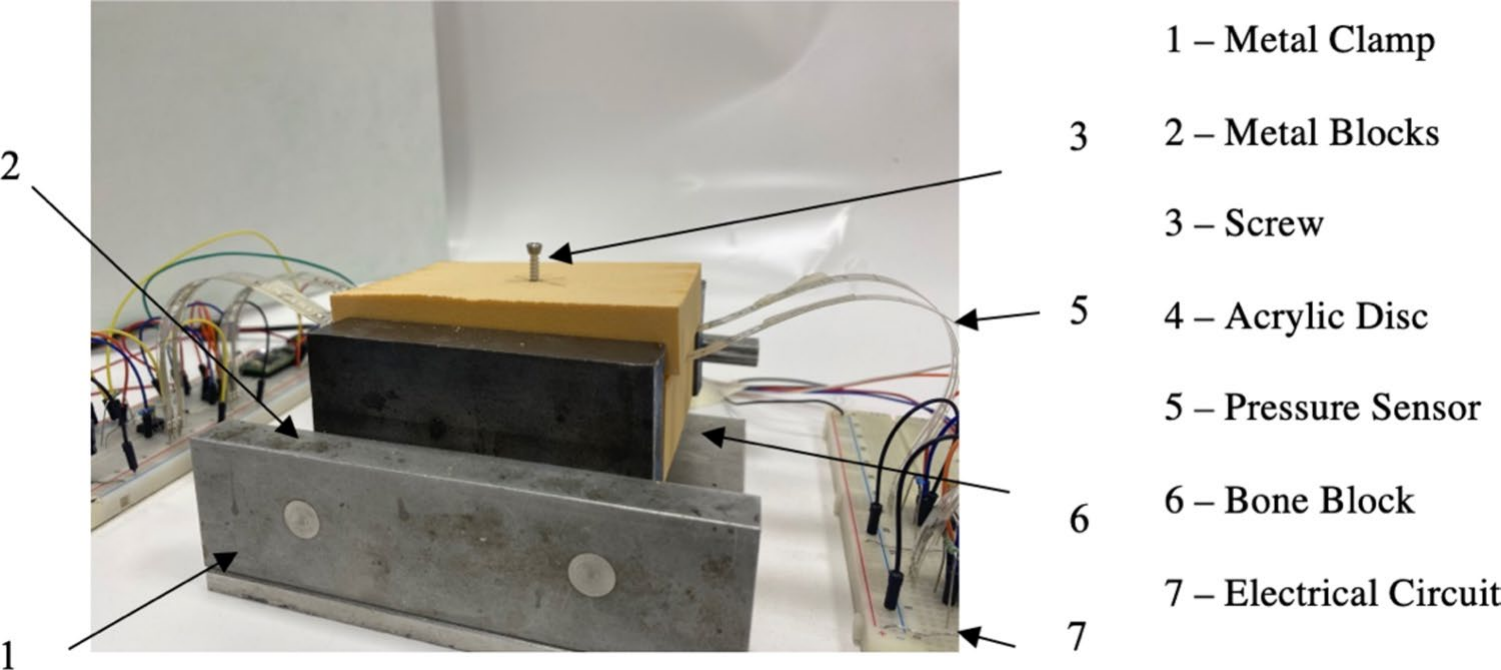
The final experimental set up of the foam block including the electrical circuit supporting the FlexiForce sensors

Four FlexiForce sensors were set up and programmed by a specialist sensor technician in a parallel circuit. The sensors act as a variable resistor and the resistance is changed by an applied force. The circuit will convert the resistance into voltage, which can be converted into force. These sensors were chosen for the thin width (0.3 mm) in order to maximise the contact between the opposing surfaces. A metal tabletop block was used to restrict twisting of the individual blocks during fastening and ensure frictionless movement of the blocks, as illustrated in Fig. 1.

**Fig. 2:**
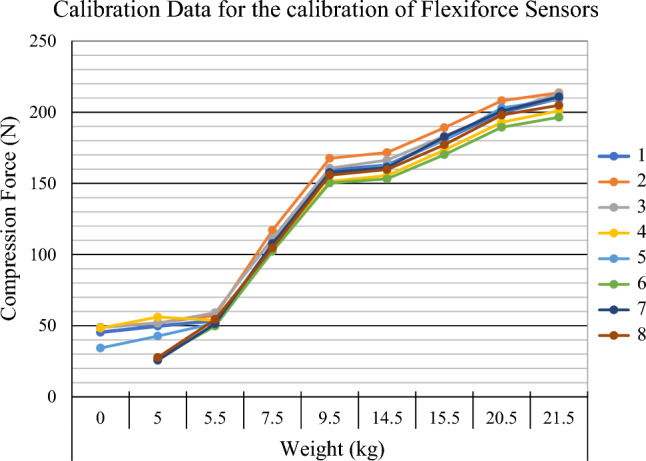
Calibration graph illustrating the output measured by the FlexiForce sensors in comparison with a known weight (kg)

Prior to conducting the study, the sensors were calibrated up to 215 N to determine their accuracy. In order to improve the calibration and accuracy of the sensor, a 0.5-mm acrylic disc was placed on top of each sensor to ensure maximal force was detected by the sensor and ensuring minimal dissipation to the sensors surrounding. The sensors were reset, and calibration was repeated between each new screw in order to ensure the sensors remained accurate throughout the study. The final accuracy of the sensors was ± 10 N up to a maximum force of 215 N (Fig. 2).

#### Fixation procedure

A simulated transverse fracture was created with a fracture gap of 0.8 mm, which is within the range of previous studies that have outlined the impact of fracture gap as a variable affecting the compression force generated by the screw [1]. All the screws were 30 mm in length in order to ensure they would traverse the simulated fracture gap. Four FlexiForce sensors recorded the compression forces (Newtons-N) between the two articulating surfaces of the bone blocks as each screw was tightened. Each sensor was placed close to the screw during fastening in order to improve the accuracy of detection and to measure forces acting in multiple directions. Each block was pre-drilled according to the manufacturer’s instructions.

Each screw was tightened by the surgeon through tactile feedback to mimic their application in a clinical setting. Compression force (*N*) was measured after every 180° revolution of the screw during fastening and recorded after waiting for 10 s in order to allow the sensors to stabilise without the additional force of the surgeon and the screwdriver. Measurements were recorded once the screw head disappeared from the surface of Block A (0 revolutions) to replicate the insertion of the screw following insertion past the articular cartilage in a joint. These were recorded until the compression force did not change significantly to avoid over-fastening of the screws which reduces the compression force due to stripping bone. The mean peak compression force was calculated from a sample of 3 screws for each screw type. The process of fastening and recording results was repeated for each screw type on a separate block.

#### Statistical analysis

Statistical analysis of the results collected has been conducted using IBM SPSS Statistics (SPSS v27, IBM Corp., Armonk, New York). One-way analysis of variance (ANOVA) test with post-hoc Tukey’s analysis was conducted to compare the results of HCS with independent locking screws. Statistical significance was defined as *p* < *0.05* for all tests.

## Results

### Large locking screw versus HCS

**Fig. 3:**
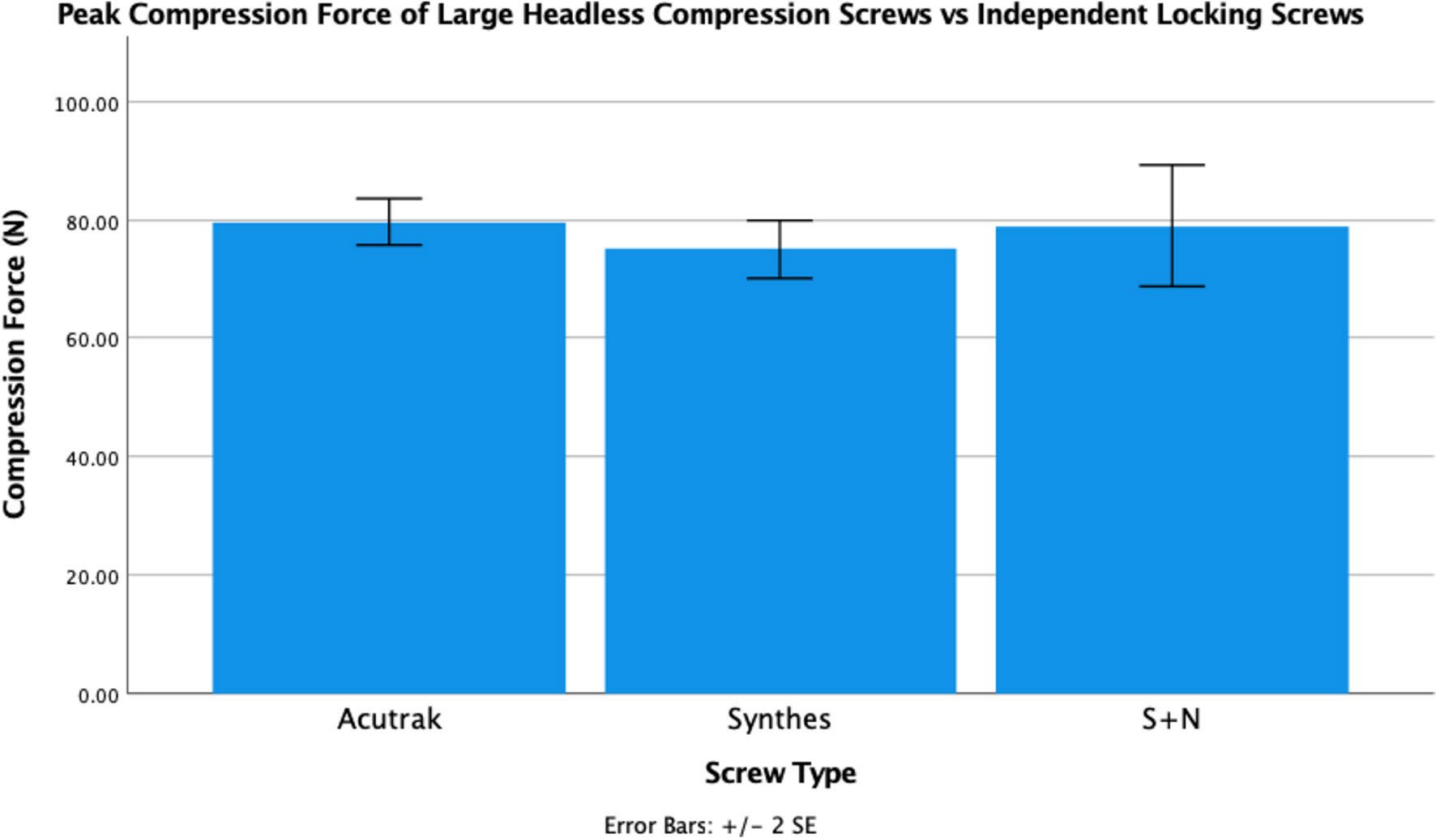
Mean peak compression force (*N*) generated by 3.5-mm headless compression screws and independent locking screws with corresponding standard error bars. There was no statistically significant difference with *p* = 0.641 for Synthes and *p* = 0.990 for Smith + Nephew (S + N)

Figure 3 demonstrates that within the large screw group, the mini Acutrak generated an overall mean peak compression force of 80 N ± 7 N, similar to the 75 N ± 8 N and 79 N ± 18 N generated by the Synthes 3.5-mm and the Smith and Nephew 3.5-mm locking screws with no significant difference noted (*p* = 0.641 and *P* = 0.990, respectively).

**Fig. 4:**
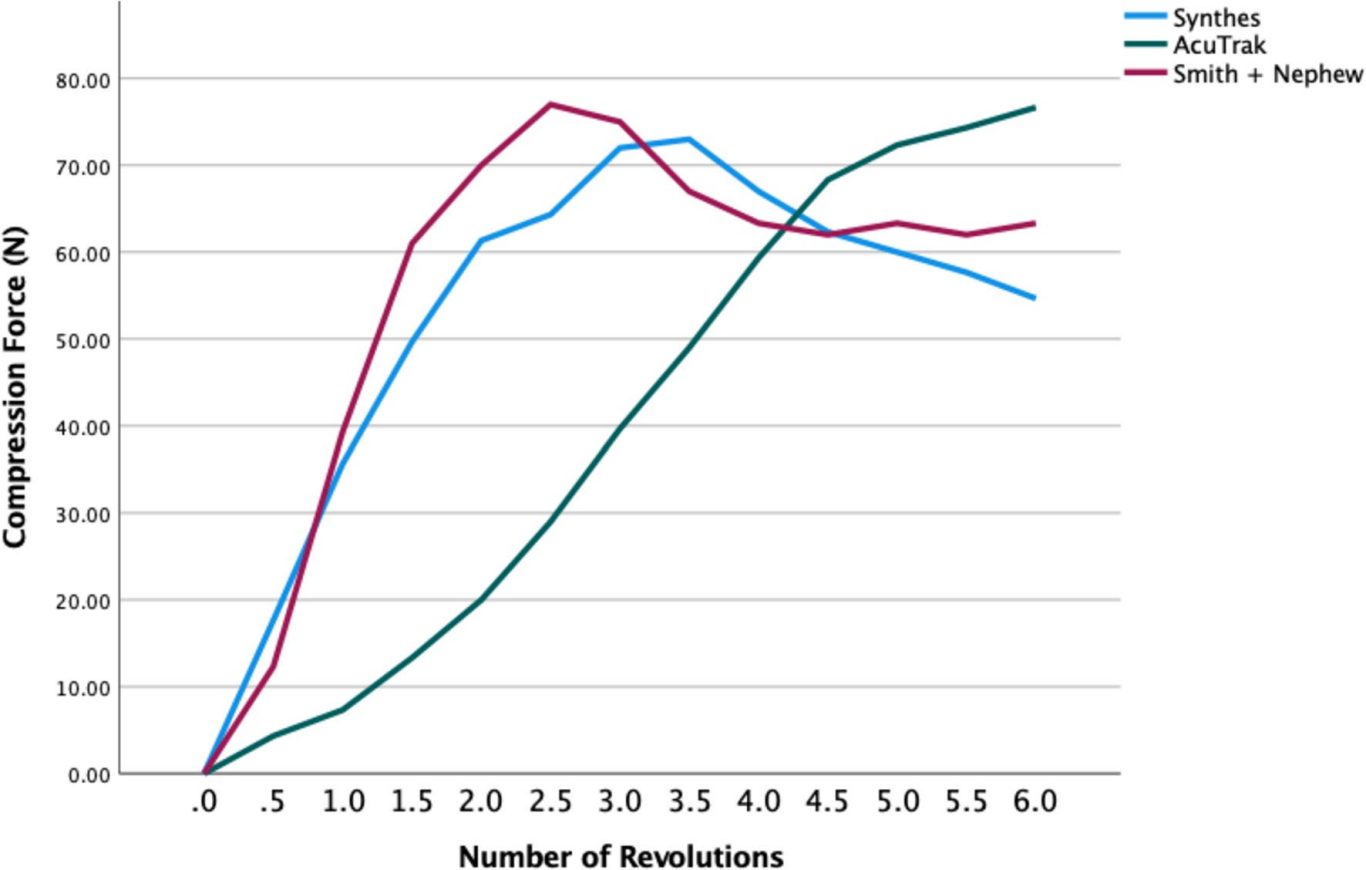
Number of revolutions taken to achieve the mean peak compression force of 3.5-mm headless compression screw (Acutrak), 3.5-mm independent locking screws (Synthes and Smith + Nephew)

Within the large screw group, the Synthes and Smith and Nephew 3.5-mm locking screws achieved their peak compression force sooner than the mini Acutrak screw (Fig. 4).

### Small locking screw versus HCS:

**Fig. 5:**
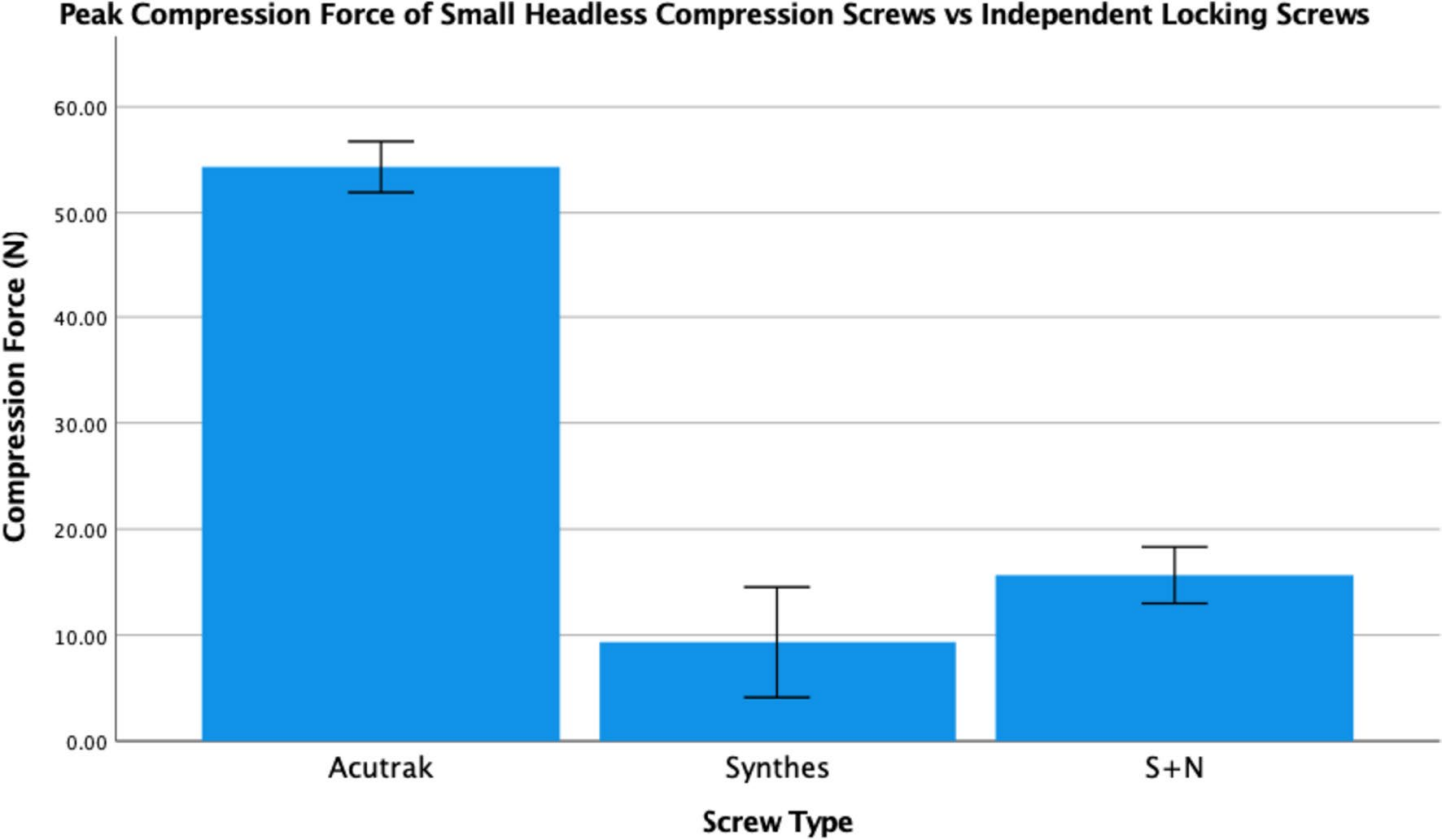
Mean peak compression force (*N*) generated by 2.8-mm headless compression screws and 2.7-mm independent locking screws with corresponding standard error bars. * Indicates statistical significance with *p* =  < 0.001 for Synthes and *p* =  < 0.001 for Smith + Nephew (S + N)

Within the small screw group (Fig. 5), the micro Acutrak generated an overall mean peak compression force of 54 N ± 4 N, compared to 17 N ± 4 N and 13 N ± 9 N for the Synthes and Smith and Nephew 2.7-mm locking screws which was significantly different (*p* = 0.000006 and *P* = 0.00001, respectively).

**Fig. 6:**
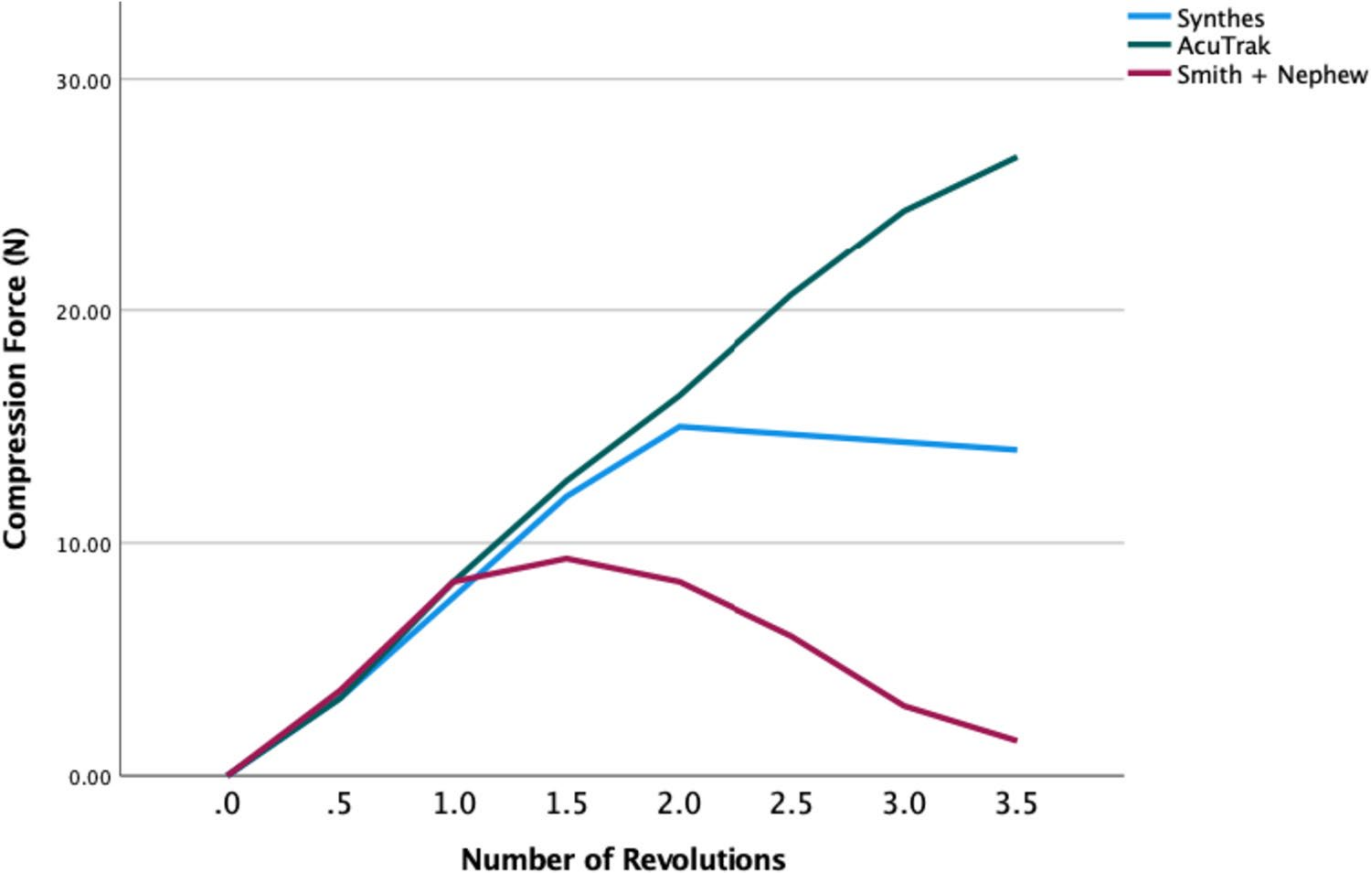
Number of revolutions taken to achieve the mean peak compression force of 2.8-mm headless compression screw (Acutrak), 2.7-mm independent locking screws (Synthes and Smith + Nephew screws)

Within the small screw group, the micro Acutrak and small locking screws had similar compressive forces until 1.5 revolutions. After 2 revolutions the mini Acutrak screws continued to increase their compressive forces whereas the locking screws did not (Fig. 6).

## Discussion

The purpose of headless compression screws was designed to provide sufficient compression without a screw head penetrating into the joint [1, 5, 6, 18]. Locking screws show similar design features and have the additional benefit of being widely available and with a large array of sizes in diameter and length as well being relatively inexpensive. The main objective of this study was to determine whether independent locking screws can generate compression forces comparable to headless compression screws. This is to determine whether they may be interchangeably used in clinical practice in the internal fixation of intra-articular fractures.

The results of this study have shown that independent locking screws do generate compression forces. This result is likely due to the design and variable thread between the head and the tail of the independent locking screw mimicking the differential thread pitch within the HCSs.

The only difference we identified was that smaller 2.5-mm-diameter locking screws achieved their peak compression after 1.5 revolutions whereas larger locking screws achieved their peak compression after 2.5 revolutions.

We do not know what compression is required in vivo and in addition fractures are usually compressed initially with a clamp prior to screw insertion. Indeed, the clinical study by Felstead et al. demonstrated that all their patients achieved successful bony union at 6 months postoperatively and all screws remained in position with no displacement [12].

The methodology of this study was informed by previous biomechanical HCS studies and has produced the results consistent with their studies. Grewal et al. reported a mean peak compression of 68.6 N ± 36.4 N for Acutrak using a similar experimental set-up [18]. Beadel et al. reported a mean peak compression force of 92 ± 56 N for Mini Acutrak screws [3]. In our study, we maintained a short fracture gap (0.8 mm) to ensure that the impact of fracture gap was limited from affecting the results. Previous studies have shown that Acutrak screws will generate less compression force with larger fracture gaps [1, 6, 8].

The effect of pre-drilling has been previously studied by Assari et al. [1], which has shown that there is very little significance between pre-drilling and relying solely on the self-cutting ability of the screw during fixation. For this study, it was crucial to pre-drill prior to screw fixation according to the head pitch diameter and tail pitch diameter for the top and bottom blocks, respectively, in order to prevent fracture gapping as the foam was of a uniform density. In clinical practice, this would not be an issue due to the thin depth of the subchondral bone and metaphyseal bone within the articulating bone.

## Study limitations and future work

One limitation of this study was the use of polyurethane foam models. These models were used for their similar densities to cancellous bone and have consistent porosity, which was advantageous for reproducibility [1]. However, peri-articular bone has a mixture of hard subchondral bone as well as cancellous bone. Previous studies have also acknowledged this as a limitation of bone model studies [1, 8].

Another limitation of the study when making clinical inferences is related to the use of a static environment which does not include the effect of bone healing and cyclical loading or the effect of multiple screws and screw trajectory. Gruszka et al. [16] concluded that there was no statistical difference in a study comparing cyclical loading of HCS but further evaluation would need to be done to look at displacement following locking screw use as headless compression screws.

Previously, Felstead et al. [12] demonstrated that independent locking screws have proven to be successful alternative to HCSs in the context of elbow fractures. All patients achieved successful bony union at 6 months postoperatively and all screws remained in position with no displacement [12]. This study supports the use of locking screws as an alternative to headless compression fractures in intra-articular fractures and supports this biomechanical study [12]. The increased inventory and availability of locking screws make them favourable during complex cases. Figures 11–13 illustrate clinical examples of complex cases that the senior author (EG) has used locking screws to manage. It would be beneficial to conduct further clinical trials on a larger patient population across a wider variety of intra-articular fractures to explore their longevity in situ.

It was interesting that independent locking screws achieved peak compression force in fewer revolutions in comparison with HCS. The loss of compressive force after 1.5 revolutions in the 2.5-mm locking screw group may be related to the effect of pre-drilling and it would be useful to repeat this study using different drill diameters. For that reason, we would only support the use of 3.5-mm locking screws; however, clinically we do not know the number of revolutions required and the total compression required for fracture stability and union.

In addition, previous studies [1] have shown that torque is a misleading factor in the generation of compression force and it would be beneficial to conduct a quantitative study with compression force and torque generated by the independent locking screw to determine whether the fastening torque as applied by the surgeon is an indicator for the loss of compression force [19]. Furthermore, it would inform the surgeon during training and when deciding between an independent locking screw and HCS in clinical practice.

## Conclusion

In conclusion, locking screws are capable of generating compression forces. Peak compression is comparable in the larger screw group whereas in the smaller screw group locking screws generated similar compression to HCS initially and then tailed off. This is of uncertain clinical relevance. Peak compression forces of locking screws are generated with fewer revolutions in comparison with the headless compression screws.

The relevance of this study to clinical practice is that the locking screws tend to be more readily available with a larger inventory and lower costs and supports the clinical study by Felstead et al. which demonstrated the favourable results using independent locking screws as headless compression screws in elbow fractures.

### Supplementary Information

Below is the link to the electronic supplementary material.

Supplementary file1 (DOCX 601 KB)

Supplementary file2 (DOCX 233 KB)
