# Supplementary material for: Biomechanical comparison of headless compression screws versus independent locking screw for intra-articular fractures
Source: Eur J Orthop Surg Traumatol. 2023 Dec 19;34(3):1319–25. doi: 10.1007/s00590-023-03792-8 (PMC10980631; doi:10.1007/s00590-023-03792-8)
Supplement: Supplementary file 1 — Supplementary file1 (DOCX 601 KB) [file 590_2023_3792_MOESM1_ESM.docx]

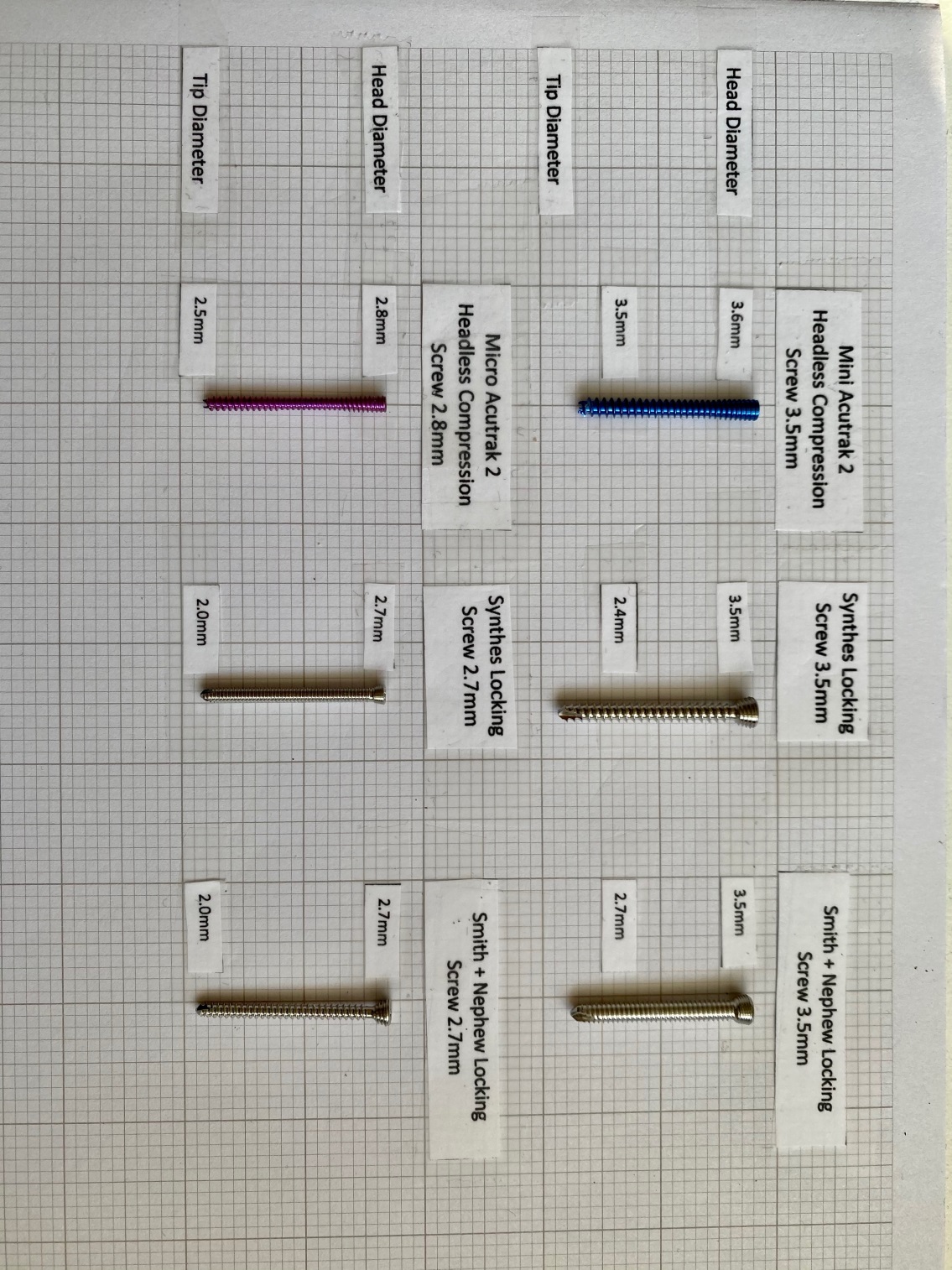


*Suppl 1: Summary Image of Headless Compression Screws (Mini and Micro Acutrak 2) and Independent Locking Screws (Synthes and Smith and Nephew). The corresponding dimensions have been outlined above. All screws are 30mm in length.*
