# Supplementary material for: Biomechanical comparison of headless compression screws versus independent locking screw for intra-articular fractures
Source: Eur J Orthop Surg Traumatol. 2023 Dec 19;34(3):1319–25. doi: 10.1007/s00590-023-03792-8 (PMC10980631; doi:10.1007/s00590-023-03792-8)
Supplement: Supplementary file 2 — Supplementary file2 (DOCX 233 KB) [file 590_2023_3792_MOESM2_ESM.docx]

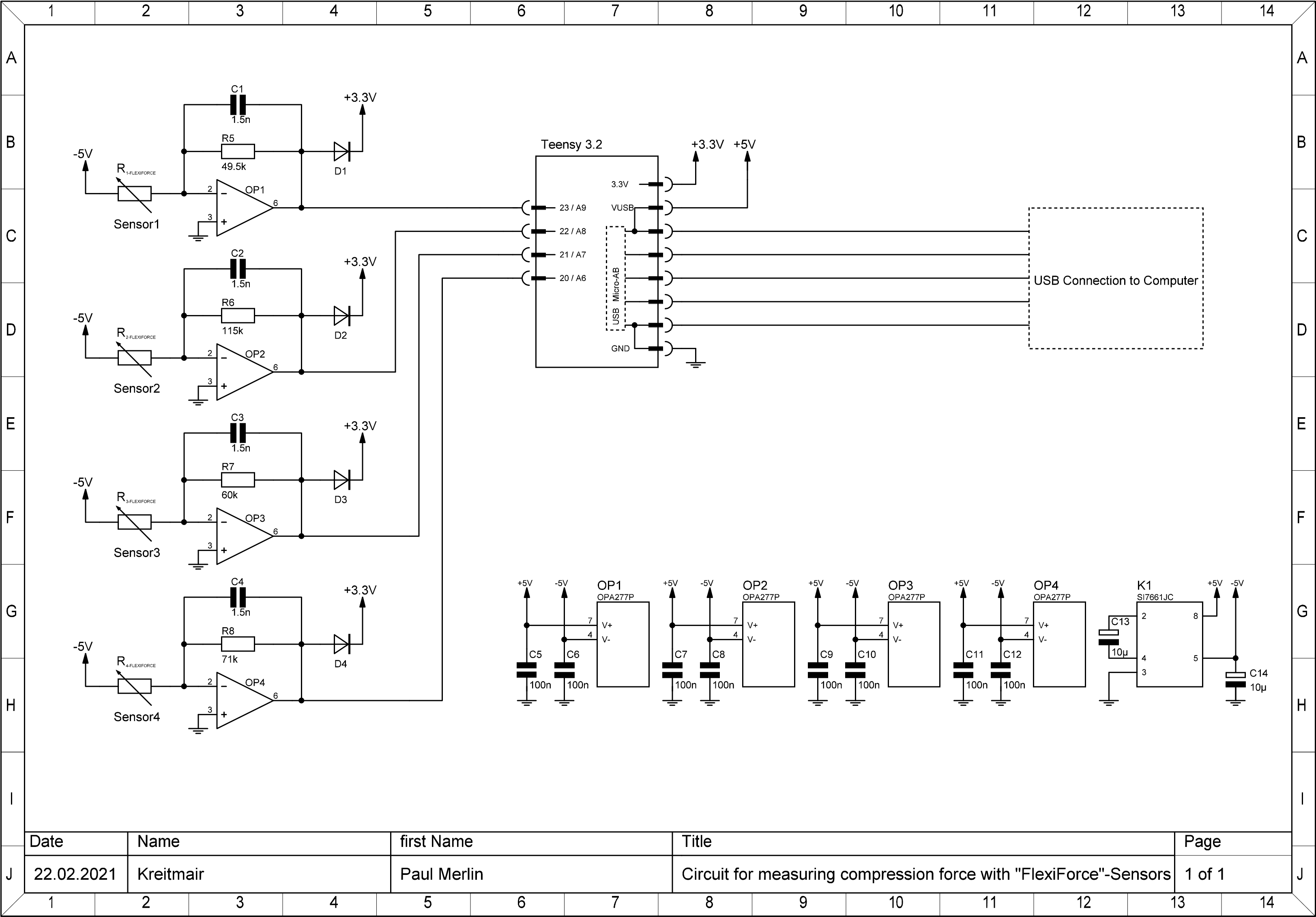


*Suppl 2: Schematic diagram illustrating the supporting circuit board for the Flexiforce sensors used in this study.*
